# *Lupinus albus* γ-Conglutin, a Protein Structurally Related to GH12 Xyloglucan-Specific Endo-Glucanase Inhibitor Proteins (XEGIPs), Shows Inhibitory Activity against GH2 β-Mannosidase

**DOI:** 10.3390/ijms21197305

**Published:** 2020-10-03

**Authors:** Stefano De Benedetti, Elisabetta Galanti, Jessica Capraro, Chiara Magni, Alessio Scarafoni

**Affiliations:** Department of Food, Environmental and Nutritional Sciences, Università degli Studi di Milano, 20133 Milano, Italy; stefano.debenedetti@unimi.it (S.D.B.); elis.galanti@gmail.com (E.G.); jessica.capraro@unimi.it (J.C.); chiara.magni@unimi.it (C.M.)

**Keywords:** seed proteins, γ-conglutin, enzyme inhibitors, plant cell wall-degrading enzymes, plant defense, *Lupinus albus*

## Abstract

γ-conglutin (γC) is a major protein of *Lupinus albus* seeds, but its function is still unknown. It shares high structural similarity with xyloglucan-specific endo-glucanase inhibitor proteins (XEGIPs) and, to a lesser extent, with *Triticum aestivum* endoxylanase inhibitors (TAXI-I), active against fungal glycoside hydrolases GH12 and GH11, respectively. However, γC lacks both these inhibitory activities. Since β-galactomannans are major components of the cell walls of endosperm in several legume plants, we tested the inhibitory activity of γC against a GH2 β-mannosidase (EC 3.2.1.25). γC was actually able to inhibit the enzyme, and this effect was enhanced by the presence of zinc ions. The stoichiometry of the γC/enzyme interaction was 1:1, and the calculated *K*_i_ was 1.55 μM. To obtain further insights into the interaction between γC and β-mannosidase, an in silico structural bioinformatic approach was followed, including some docking analyses. By and large, this work describes experimental findings that highlight new scenarios for understanding the natural role of γC. Although structural predictions can leave space for speculative interpretations, the full complexity of the data reported in this work allows one to hypothesize mechanisms of action for the basis of inhibition. At least two mechanisms seem plausible, both involving lupin-γC-peculiar structures.

## 1. Introduction

Glycoside hydrolases (GHs) are enzymes that catalyze the hydrolysis of the glycosidic linkage of glycosides [1]. The GHs have been classified into more than 100 families [2]. Each family contains proteins that are related by sequence and, in consequence, share common structural properties [3]. The classification of GH families into larger groups, termed “clans”, has been proposed [4,5]. A clan is a group of families that possess significant similarity in their tertiary structure, catalytic residues and mechanisms of action [6]. Thus, knowledge of three-dimensional structure and the functional assignment of catalytic residues is required for classification into clans. The various biological functions of GHs are several and include the degradation of different plant cell wall polysaccharides [7,8]. The primary walls of plant cells are essentially composed of cellulose, pectin and combinations of hemicelluloses [9]. An important hemicellulose in most plants is xyloglucan, but glucuronoxylan, arabinoxylan, glucomannan and galactomannan are also found in different proportions in the primary and secondary walls of many botanical families [10]. Galactomannans are found as a major component of the endosperm cells in the seeds of several Leguminosae, including lupin [11,12].

Some pathogenic microorganisms are able to secrete GHs to penetrate plant cell walls [13]. As a response, plants produce GH inhibitor proteins (GHIPs) [14].

γ-conglutin (γC) accounts for about 4% of total seed protein, which contributes 35–40% to the dry seed weight of the leguminous plant *Lupinus albus* [15]. In the last decade, this protein has been receiving increasing interest because of its demonstrated capacity to lower blood glucose levels in humans and animals when orally administered [16,17]. This allows one to hypothesize its use as an agent for the treatment of patients suffering from prediabetes [18].

All of this aside, the natural biological role of γC is still far from clear. Although it has been considered, for a long time, a classical seed-storage protein, more recent studies broaden its functions to include a possible role in defense against pathogenic microorganisms [19,20].

γC is a homo-hexameric glycoprotein, in which each monomer of 45 kDa is made up of two disulfide-bonded polypeptides of about 29 and 17 kDa [15,21] that originate from a single precursor protein synthesized during seed development and processed by post-translational proteolysis [22]. The protein is glycosylated in the 29 kDa subunit [15,23]. The protein undergoes association–dissociation transition between the hexameric and monomeric forms according to the pH conditions. The monomeric form predominates at slightly acidic pHs [24]. Although γC is stored in the cotyledon protein bodies of mature quiescent seeds, the protein has been detected in the extracellular apoplastic regions of germinating seeds [25]. In addition, γC is able to bind divalent metal ions, especially Zn^2+^ and Ni^2+^ [26], and phospholipids [27].

Two genes encoding γC have been identified in *Lupinus albus*, but only one is quantitatively expressed and accumulated in the developing seeds [22,28].

γC shares high structural similarities with two families of GH inhibitors, namely, xyloglucan-specific endo-β-1,4-glucanase inhibitors (XEGIPs) and *Triticum aestivum* endoxylanase inhibitors (TAXI-I). While XEGIPs inhibit the hydrolytic activity of a xyloglucan-specific β-1, 4-endo-glucanase (XEG) isolated from *Aspergillus aculeatus* and belonging to the GH12 family [29,30], TAXI-Is are inhibitors of GH11 members [31]. γC lacks the typical inhibitory activity against representative fungal GH11, GH12 and polygalacturonase, although its de novo expression can be elicited by chitosan [19,20].

XEGIPs have been found to be widespread in dicots. They have been detected in the medium of cultured tomato cells [30] and carrot calli [32], and isolated from the nectar of ornamental tobacco [33]. Moreover, it has been demonstrated they that are capable of protecting potatoes from disease caused by *Phytophthora infestans* [34], and they were found to be up-regulated in apples in response to infection by *Botryosphaeria dothide* [35] and in *Humulus lupus* [36]. In cereals, three types of GHIPs occur in a fairly coordinated fashion throughout grain development and germination, the *Triticum aestivum* L. endoxylanase inhibitors (TAXI-I-like) being the most represented [31,37].

Sequence alignments and structural studies showed that in XEGIPs and the TAXI-I protein, two functional domains are responsible for the inhibitory capacity [21,38]. Both are located in the C-terminal regions of the proteins. The first is located between cys10 and cys11, and defines a surface-exposed region known as inhibitory loop 1 (IL1), where a conserved arginine in XEGIP-like proteins, or a conserved leucine in TAXI-I, are involved in binding with the respective GH12 or GH11. In γC, instead, IL1 is missing due to a deletion of about five amino acids [19,21]. This is likely the cause of an unfavorable local spatial conformation of the protein for the correct interaction with the enzyme [20], leading to the lack of inhibitory capacity of γC and other similar legume proteins, including soybean Bg7S [39]. The disulfide bridge between cys9 and cys12 defines another functional region called inhibitory loop 2 (IL2), where the key amino acids are an arginine residue in XEGIPs or a histidine in TAXI-I and γC. The sequence of the IL2 loop of γC is more similar to the sequence of the IL2 loop of TAXI-I than to the one of XEGIPs. Studies on γC mutants [20] demonstrated that the presence of IL1 is not strictly required to manifest inhibition, even if the inserted amino acid stretches enhanced the activity. Moreover, the structure of IL2 is the essential element that is very likely necessary not only to manifest the inhibitory competence but also to drive the specificity toward the respective target GH.

In the present work, we undertook lab experiments and in silico predictions aiming to define and characterize the inhibitory activity of γC extracted from *L. albus* seeds. γC was tested against a GH2 β-mannosidase (EC 3.2.1.25) and a GH5 xyloglucan-specific endo-β-1,4-glucanase (EC 3.2.1.151).

## 2. Results and Discussion

**Figure 1:**
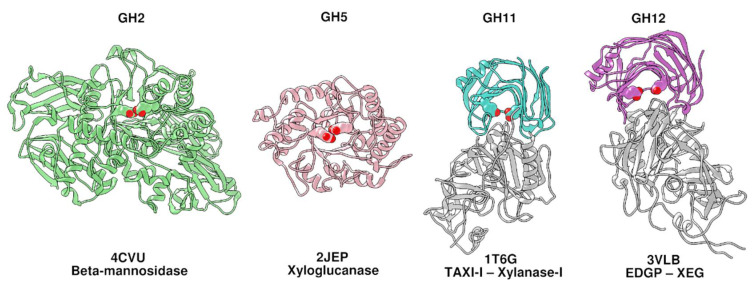
Structure comparison of representative members of GH families. For each structure, the PDB code and the enzyme names are reported below each model. GH11 and GH12 are reported with the respective inhibitors (gray) as they appear in PDB accessions. Catalytic amino acids are shown in spacefill representation.

Enzymes whose activity could be influenced by γC have been elusive for a long time [19]. A great number of findings indicate that the target of γC is almost certainly a GH enzyme. In previous work, the possible inhibitory activity of γC was tested unsuccessfully against GH11 and GH12 [19,20], all included in GH clan C [4]. Despite their low sequence identity (only the three amino acids essential for catalysis are completely conserved across all members [40]), the overall three-dimensional structures for all the known GHs grouped in this clan are remarkably similar (Figure 1, cyan and purple models). Conversely, in the present work, we focused our attention on enzymes having different structural features than GH11s and GH12s, not yet explored for their susceptibility to γC inhibition. The choices were a β-mannosidase (GH2) and a xyloglucan-specific endo-β-1,4-glucanase (GH5), both members of GH clan A. The catalytic domain of the enzymes from this clan has a (β/α) 8-barrel fold, also called a TIM-barrel fold [4]. The first enzyme [41] was selected because β-(1→4)-linked polysaccharides containing mannose are major components of the endosperm cell walls in seeds of several legume plants, including soybeans and lupins [11]. We thus hypothesized that a potential inhibitory activity from the lupin’s γC could be exerted towards enzymes targeting the major constituent of its cell wall. The second enzyme was considered because the described substrate specificity is similar to that of the GH12 xyloglucan-specific endo-glucanase [29,42], against which carrot EDGP and tomato XEGIP are active as inhibitors [30,39]. From the structural point of view, the selected enzymes are very different from GH11 and GH12 enzymes, but similar to each other (Figure 1, green and pink models).

**Figure 2:**
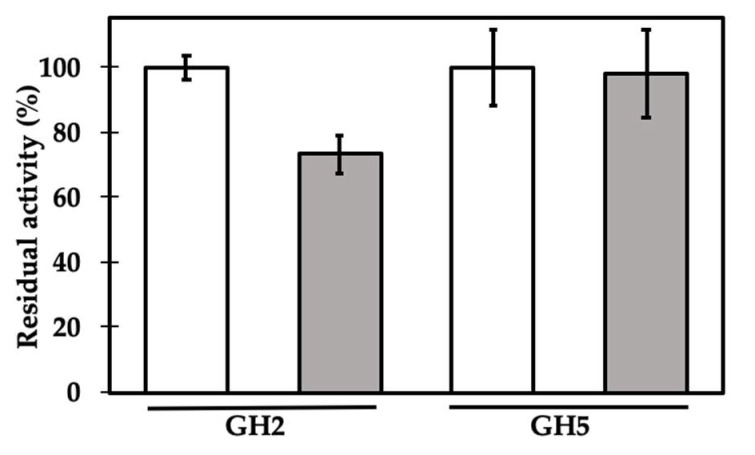
Enzyme residual activity in the absence (white bars) and in the presence of γC at a molar ratio of 1:1 (gray bars). GH2 and GH5 stand for β-mannosidase from *Cellulomonas fimi* and for xyloglucan-specific-endo-β-1,4-glucanase from *Paenibacillus polymyxa*, respectively. Residual activity was calculated as (AE-AEI)/AE × 100, where AE is the measured enzyme activity (mmol/min) and AEI is the activity of the enzyme in the presence of γC. See text for experimental details. Each point is the mean of three assays.

GH2 β-mannosidase from *Cellulomonas fimi* [43] and GH5 xyloglucan-specific endo-β-1,4-glucanase from *Paenibacillus* sp. [44] activities were assayed by incubating each enzyme with chromogenic substrates, as described in Materials and Methods, in the presence or absence of γC, at an initial molar ratio of 1:1 (Figure 2). Only in the first case did the presence of the lupin protein reduce the enzyme activity, to about 73% of its maximum activity, in the adopted experimental conditions, whereas no significant effects were observed when it was tested against the GH5 enzyme.

This is the first time we have been able to describe an inhibitory activity for *L. albus* γC. Encouraged by this result, we focused on the further characterization of GH2 β-mannosidase’s inhibiting activity.

**Figure 3:**
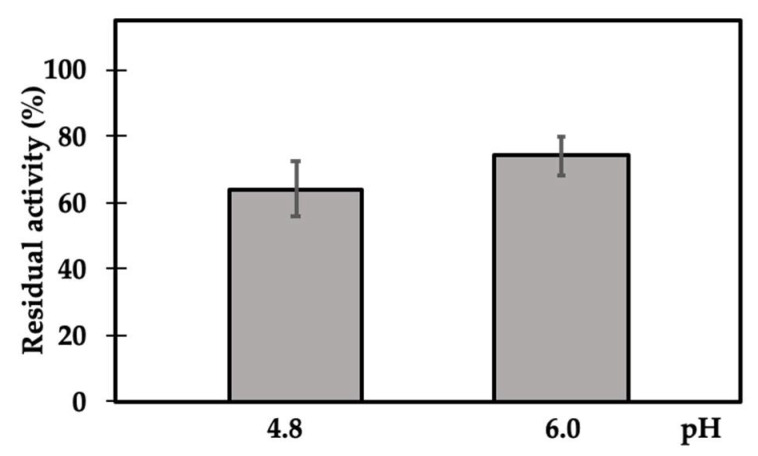
Residual activity of GH2 β-mannosidase incubated in the presence of γC at different pHs, using pNP-β-d-mannopyranoside as the substrate. Activities determined at pH 4.8 and 6.0 were not statistically different (*p* ≤ 0.05). Residual enzyme activity is expressed as percentage activity compared with the enzyme alone. Each point is the mean of three assays.

At first, the inhibitory activity was tested at two different pHs, regardless of the optimal enzyme reaction conditions. The native quaternary structure of γC is determined by the transition from a hexameric to monomeric state, according to the acidity of the medium [24]. The results reported in Figure 3 show β-mannosidase’s residual activity at pH 4.8 (where γC is completely in the monomeric form) and pH 6.0 (within the optimal range for enzyme activity and where the γC monomeric form disappears to form oligomers). The residual activities observed at pH 4.8 and 6.0 were very similar, and small variations may depend on the oligomerization state of γC or the different charges that proteins assume at different pHs. However, the observed differences were not statistically different (*p* ≤ 0.05).

**Figure 4:**
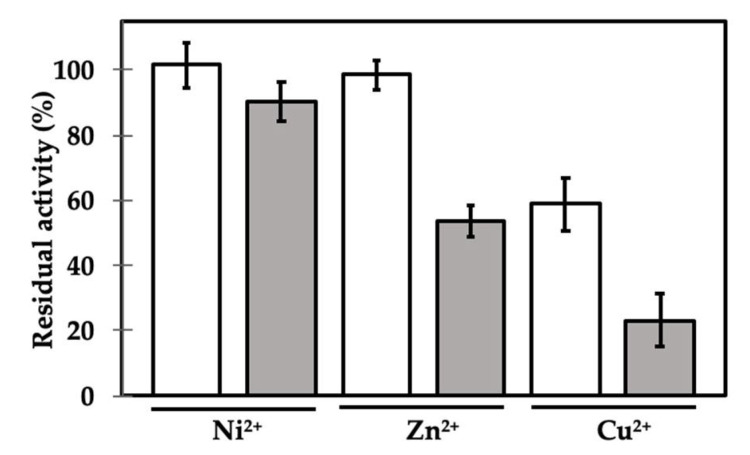
Incubation of GH2 β-mannosidase in the absence (white bars) and in the presence of γC at a molar ratio of 1:1 (gray bars), with 1 mM Ni^2+^, Zn^2+^ and Cu^2+^. Residual enzyme activity is expressed as percentage activity compared with the enzyme alone by using pNP-β-d-mannopyranoside as the substrate. Each point is the mean of three assays.

We then tested the effects of three metal ions (Cu^2+^, Ni^2+^ and Zn^2+^) that have been previously shown to produce controversial effects on the GH2 enzyme’s activity. It has been indeed shown that many mannosidases, including those belonging to the GH2 group, may be sensitive to metal ions. The results, however, are debated, since the same ion can positively or negatively influence the activity of different enzymes [45,46,47,48]. Intriguingly, γC was shown to be able to interact with metal ions, in particular, those investigated [26]. The presence of the ions, in most cases, led to a decrease in the solubility of the protein, when present in molar excess and according to the pH of the incubation buffer. However, 1 mM Zn^2+^ did not affect the solubility of the protein at pH values between 4.5 and 6.3 [26]. We therefore incubated the β-mannosidase with or without γC, in the presence of different metal ions, at the final concentration of 1 mM (Figure 4).

The presence of Ni^2+^ and Zn^2+^ ions alone did not affect the activity of β-mannosidase in the adopted experimental conditions, whereas Cu^2+^ markedly decreased its functionality. When γC was added, the residual activity was lower in all cases. The activity in the presence of Ni^2+^ was only slightly affected, but it dropped drastically when Zn^2+^ was added (about 55%). Thus, the presence of Zn^2+^ increases the inhibitory activity of γC by about 20%. The metal ion could possibly be coordinated by specific amino acid residues promoting conformational changes in γC regions involved in the interaction with the enzyme, such as the inhibitory loop IL2, and, in turn, favoring the inhibitory competency (Supplementary Figure S1). The results obtained with Cu^2+^ ions are difficult to interpret, since the enzyme itself is very sensitive to its presence in the incubation medium. It is worth noting that the purified γC used throughout the experiments described here did not carry any kind of metal ions, as confirmed by inductively coupled plasma (ICP)-MS.

Similarly, as reported for GH2 enzymes, the effect of metal ions on GH11 and GH12 seems not to follow a general rule. A GH11 enzyme from *Aspergillus tamarii* was strongly inhibited by 5 mM Cu^2+^ and Zn^2+^, while Ni^2+^ increased enzyme activity [49]. Rawat et al. [50], instead, reported on a GH12 endo-glucanase from *Aspergillus niger* strongly inhibited by Cu^2+^, while the Zn^2+^ inhibitory effect was only partial at the same concentration. The activity of a GH12 acidic endo-glucanase from *Gloeophyllum trabeum* was not affected by Zn^2+^ and Cu^2+^, even at 50 mM concentrations [51]. Juturu and Wu [52] reviewed the effects of metals on xylanases belonging to different families (GH10, GH11 and GH39), all negatively affected by Hg^+2^, Fe^+2^, Co^+2^, Mn^+2^, Ag^+2^, Pb^+2^ and Cu^+2^, but they provided no clues about Zn^2+^ and Ni^2+^. Testing the effect of a possible inhibitory activity of γC against GH12 or GH11 enzymes in the presence of metal ions was out of the scope of this work.

**Figure 5:**
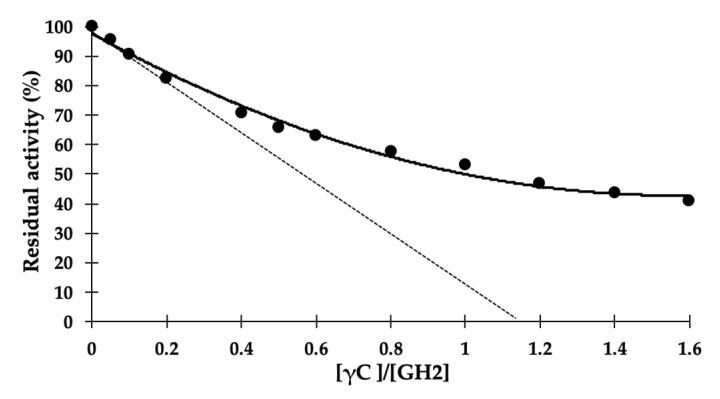
Titration curve of GH2 β-mannosidase with γC. Increasing concentrations of γC were added to a fixed concentration of enzyme (11.8 μM). Residual enzyme activity is expressed as percentage activity compared with the enzyme alone, using pNP-β-d-mannopyranoside as the substrate (0.8 mM), at pH 6.0, in the presence of 1 mM Zn^2+^. Each point is the mean of three assays. Error bars have been omitted for better clarity. The best fit curve (black full line) has R^2^ = 0.9913. The x-intercept value of the traced tangent line (dash line) indicates an inhibitor/enzyme stoichiometry of 1.14.

We finally aimed to determine some kinetic parameters, in particular, the *K*_i_, of the Zn^2+^-mediated γC inhibition of GH2, since this ion was the most effective in enhancing the inhibitory activity. The inhibition features were determined as usual by monitoring the hydrolysis of the chromogenic substrate pNP-β-d-mannopyranoside, this time with increasing concentrations of γC, in the presence of 1 mM Zn^2+^. The experimental data are plotted in Figure 5. The x-intercept value of the traced tangent line of the best fit curve indicated an enzyme/inhibitor stoichiometry of 1.14, a value very close to a theoretical enzyme/inhibitor ratio of 1:1.

*K*_i_ was estimated according to Cer et al. [53], who proposed a method to calculate *K*_i_ values from experimentally determined *IC50* values for enzyme inhibitors and for binding reactions between macromolecules, including proteins and ligands. The *IC50* was determined according to the equation of the best fit curve of Figure 5 (y = 22.342x^2^ − 70.059x + 97.699). The half of the maximum enzyme activity in the adopted experimental condition was reached when the molar ratio [γC]/[GH2] was 0.99. The resulting *K*_i_ was 1.55 ± 0.08 μM, assuming a competitive mechanism of action of the inhibitor. This inhibition mechanism seems the most plausible, given the mechanism of action of the other GH inhibitors [30,54] and in light of the in silico predictions and analysis described below.

To obtain further insights and to outline the possible rationale behind the interaction between γC and β-mannosidase, an in silico structural bioinformatic approach was followed. To this purpose, an *L. albus* γC 3D model was created using, as a template, the crystal structure of γC from *L. angustifolius* (PDB: 4PPH) [21], which shares 88.73% sequence identity. The predicted structure is a homo-hexamer, like the template, with an expected accuracy value (global model quality estimation (GMQE); range 0–1) of 0.82 and a qualitative model energy analysis (QMEAN) Z-score of −1.08, indicating a good agreement between the computed model structure and structures of similar sizes experimentally determined. The structure of each monomer highly overlaps the structure of γC from *L. angustifolius* with a root mean square deviation (RMSD) of 0.152 Å; the lowest homology is in the loop between amino acids 255 and 272, unmodeled in the template, containing the cleavage site of the precursor that originates the large and small subunits of γC.

**Figure 6:**
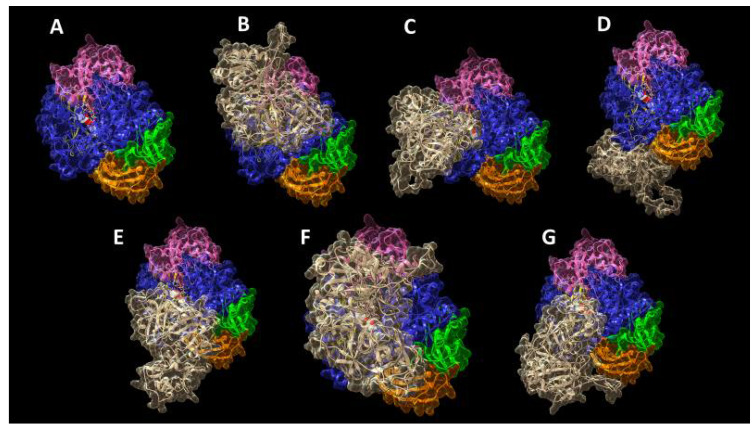
Docking results from all the tested software. ThMan2A catalytic domain is colored in blue, whereas structural domain 1 is pink, domain 2 is yellow, domain 4 is orange and domain 5 is green [55]. Catalytic amino acids E489 and E594 are represented as white spheres, and the substrate β-galactomannan is represented as white sticks. Refer to text for ThMan2A coloring details. *L. albus* γC is represented in tan color. Panel (**A**) shows ThMan2A alone; the others show the complex with γC as predicted by the pyDockWEB (**B**), ClusPro 2.0 (**C**), PRISM 2.0 (**D**), GRAMM-X (**E**), HDOCK (**F**) and FRODOCK (**G**) software. Atoms of the catalytic residues in the active site are white colored in spacefill representation to highlight the localization of the acid-base E489 and the nucleophile E594 [55].

We performed all the following in silico analyses using the structure of the homolog GH2 enzyme from *Trichoderma harzianum* (ThMan2A) [55], since the 3D structure of the GH2 β-mannosidase from *Cellulomonas fimi* used for our wet experiments has been not yet determined. The superposition of the ThMan2A structure (PDB: 4CVU) with a 3D predictive model, created on purpose with SwissModel [56] using the *Cellulomonas fimi* GH2 β-mannosidase sequence (UniprotKB: Q9XCV4) [43] as the query, indicated an excellent agreement between the two models (RMSD = 0.987 Å) (Supplementary Figure S2). Thus, we decided to perform docking analyses assigning the ThMan2A structure as the receptor and the monomer of the *L. albus* γC model as the ligand molecule, where necessary, without any restraint, following each program’s default input instructions. The monomer of γC was chosen as a docking molecule. The results are reported in Figure 6 and are relative to the best docking obtained with each piece of software, i.e., the result showing the highest score calculated including electrostatic and van der Waals energy contributions, sorted by each computation as the top solution. For better representation and clarity, ThMan2A is represented with the coloring attributed by Nascimento et al. [55], highlighting, in blue, the catalytic domain (residues 347–737), while structural domain 1 (residues 26–221) is pink, domain 2 (residues 222–346) is yellow, domain 4 (residues 738–849) is orange and domain 5 (residues 850–942) is green. The AutoDock Vina software docked the substrate β-galactomannan (GM), the main storage polysaccharide in many legume species, in several conformations into the active site; only the one with the best docking energy is shown.

It is remarkable that five algorithms for molecular docking, out of the six tested, predict the interaction between different portions of the lupin conglutin gamma model and the ThMan2A active site region, thus indicating that access for the substrate to the catalytic residues could potentially be hindered and limited by the presence of γC. Different on-line tools identify different regions of γC as responsible for these interactions; thus, it is difficult to hypothesize a univocal potential mechanism of action only on the basis of the reported data. However, it must be noted that ClusPro 2.0 (Figure 6C), which allowed the refinement of the selection of docking structures by weighing the contribution of electrostatic and van der Waals forces, indicated residues Q341, K357 and K358 of γC as responsible for the interaction. Q341 is conserved across sequences of γC homologs in other legume species, whereas K357 and K358 are peculiar exclusively to *L. albus* γC.

**Figure 7:**
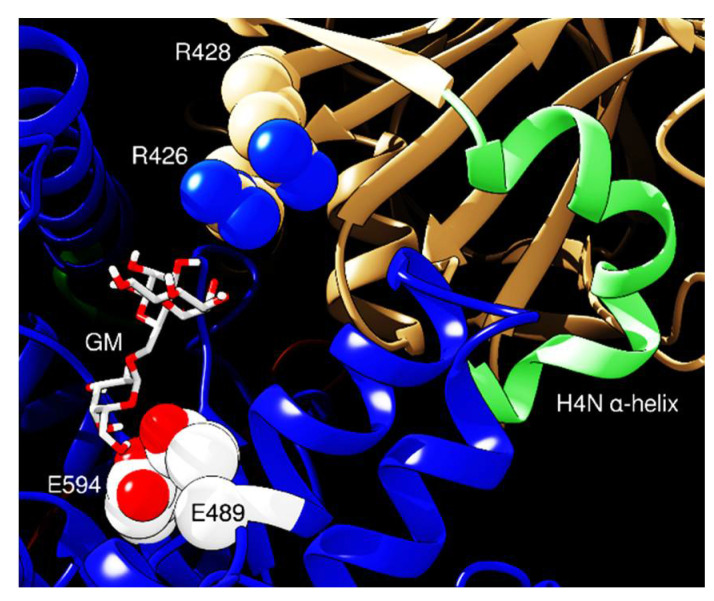
Docking of γC (tan color) and ThMan2A (catalytic domain: blue) as predicted by HDOCK software. The picture is a selected area showing the region of the active site; ThMan2A domain 1 is omitted for better representation. The banana-shaped α-helix of γC is colored in light green. Red and white balls are the catalytic residues, whereas β-galactomannan (GM) is reported as sticks.

Conversely, the analysis performed with HDOCK (Figure 7) shows a possible interaction mediated by residues R426 and R428 from γC, directed towards the ThMan2A active site. These amino acids lie within a protruding loop resembling XEGIPs’ inhibitory loop 1 IL1, in which the inhibitory activity is borne by the critical residue R322. Furthermore, XEGIPs’ inhibitory loop IL2’s competence is dependent on a conserved arginine residue (R403) [20,38]. γC, similarly to its homolog from soybeans, Bg7S, lacks these critical residues in IL1 and IL2, thus lacking inhibitory activity towards members of the GH12 family [39]. However, the R426 and R428 of γC possess the potential to mediate inhibitory activity towards members of the GH2 family. Finally, the docking analysis of the whole molecule to ThMan2A domain 3 indicates that the interaction could be mediated by helix H4N of γC, which is composed of alternate α-helical and 3_10_-helical segments and confers to this region a deformed curved shape peculiar only to γC [21]. In conclusion, despite residue R428 being conserved in all γC homologs, sequence analysis shows that R426 is conserved only in legumes and in pepper amino acid sequences [57].

**Figure 8:**
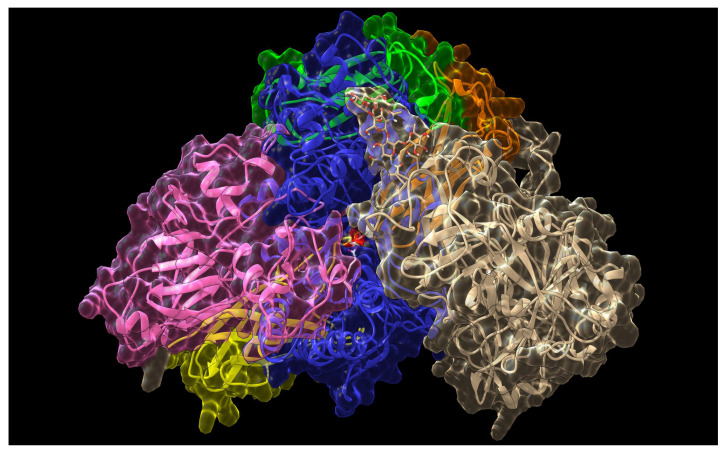
Docking prediction of γC interaction with GH2 β-mannosidase using FRODOCK software. One of the glycosylations described by Schiarea et al. [23] (Man_2_ (Fuc) GlcNAc_2_) was added to N131 with the GlyProt tool and is shown in stick representation. γC is tan colored, whereas β-mannosidase structural domains are colored as reported in Figure 6.

Intriguingly, a set of predictions made with the FRODOCK software indicated that residue N131 might lie close to the active site of the enzyme. The residue is part of the unique *N*-glycosylation consensus motif on γC. This suggests that the glycosyl moiety of γC may directly interact with the active site of the enzyme also thanks to its flexibility. It has been shown that the glycosylic portion of γC contains mannose residues at its extremity [23], thus presenting to the enzyme a structure that resembles its natural substrate (Figure 8).

The glycosyl moiety of γC lies at the surface of each monomer [21], thus making intriguing a mechanism of inhibition involving this post-translational modification. However, this hypothesis seems to be unlikely since the number of potential glycosylation sites in proteins homologous to γC is highly variable. In the EDGP sequence, indeed, there are four glycosylation consensus motifs; in NEC4, six; in XEGIP, five; and in the TAXI-I protein, only one. Glycosylation seems to have very limited effects on TAXI-I inhibitory activity [31]. However, we leave this hypothesis open and worthy of further experiments, considering that the structure of the target enzyme GH2 β-mannosidase is very different from that of the GH11 and GH12 endo-xylanases.

## 3. Materials and Methods

### 3.1. Reagents

All reagents were obtained from Sigma-Aldrich (Milan, Italy), if not otherwise specified. β-mannosidase from *Cellulomonas fimi* and xyloglucan-specific endo-β-1,4-glucanase from *Paenibacillus* sp. were obtained from Megazyme (Bray, Wicklow, Ireland), with catalog numbers E-BMOSCF and E-XEGP, respectively.

### 3.2. γ-Conglutin Purification

γC was purified from lupin seeds (*Lupinus albus*, cv. Multitalia) to homogeneity as previously described by Scirè et al. [27], lyophilized and stored at 4 °C in sealed vials. Before use, the protein was dissolved to a concentration of about 3 mg mL^−1^ in the buffer necessary for subsequent experiments. The solution was then centrifuged for 5 min at 12,000 rpm and spectrophotometrically quantified at 280 nm according to [58].

### 3.3. Enzyme Activities

GH2 β-mannosidase activity was measured in freshly prepared 100 mM sodium maleate buffer, pH 6.0, containing BSA 1 mg mL^−1^ and 80 mM pNP-β-d-mannopyranoside (Megazyme, Bray, Wicklow, Ireland), using 0.15 U of enzyme (specific activity: 13 U mg^−1^). The final volume was 3 mL. Samples were incubated at 35 °C for 15 min. The reaction was stopped using 0.5 mL of 5 M NaOH. The amounts of *p*-nitrophenol produced following enzyme activities were monitored spectrophotometrically at 410 nm. Control samples were set up by using distilled water instead of enzyme solution. Alternatively, 100 mM sodium acetate buffer, pH 4.8, was used. For inhibition assays, 0.15 U of β-mannosidase was preincubated with increasing amounts of γC in order to obtain different molar enzyme/γC ratios as indicated in the text, for 10 min at room temperature in 0.1 mL of incubation buffer. Then, the volume was adjusted to 3 mL with the same buffer containing the substrate as described above. When required, the sodium maleate incubation buffer was prepared by adding metals (ZnCl_2_, NiCl_2_ or CuCl_2_) at final concentrations of 1 mM.

GH5 xyloglucan-specific endo-β-1,4-glucanase was assayed according to [29] in 100 mM sodium acetate buffer (100 mM), pH 5.5, at 40 °C, using 2 U of enzyme (specific activity: 70 U mg^−1^) and 2 mg of beechwood xyloglucan. The final volume was 1 mL. The amount of reducing sugars produced following enzyme activity after 30 min of incubation was assessed using *p*-hydroxy-benzoic acid hydrazide according to [59]. In the inhibition assays, the enzyme was preincubated at room temperature with 150 μg of γC in order to obtain a molar enzyme/γC ratio of 1:1.

Enzyme residual activities were calculated as (AE-AEI)/AE × 100, where AE is the measured enzyme activity (mmol/min) and AEI is the activity of the enzyme in the presence of γC.

### 3.4. ICP-MS

Samples of lyophilized γC (50 mg) were dissolved in 10 mL of 65% nitric acid and digested in Teflon tubes using a microwave digestor (Anton Paar Multiwave-Eco, Rivoli, Torino, Italy). A power ramp was applied as follows: 200 W was reached over 10 min and maintained for 5 min; then, 650 W was reached over 10 min and maintained for 15 min. After a 20 min cooling time, the samples were diluted 1:40 with Milli-Q water, and the concentrations of Zn, Ni or Cu were measured by inductively coupled plasma (ICP) mass spectroscopy (Bruker AURORA M90 ICP-MS, Milan, Italy), according to [60].

### 3.5. Inhibition Data Analysis

*K*_i_ was estimated according to Cer et al. [53], using the web-server tool [61]. The input data were as follows. Substrate concentration: 800 μM; enzyme concentration: 11.8 μM; enzyme *Km*: 300 μM, according to [43]; inhibitor concentration: 11.7 μM; *IC50*: 0.99, calculated according to the equation of the best fit curve of the experimental data plotted as enzyme residual activity vs. the [γC]/[enzyme] ratio.

The stoichiometry of γC/enzyme binding was determined according to [62], tracing the tangent line of the titration curve (residual activity vs. [γC]/[enzyme]) at the higher inhibition rate point. The x-intercept value of the tangent line indicates the inhibitor/enzyme stoichiometry.

### 3.6. Sequence Searches and In Silico Predictions

The *L. albus* γC (UniprotKB: Q9FSH9I) and β-mannosidase (UniprotKB: Q9XCV4) sequences were retrieved from the UniProtKB/Swiss-Prot database (www.expasy.org).

The PDB model structure of *L. albus* γC was created using the Swiss Model homology modelling pipeline [56], a tool available on-line via the ExPASy server at https://swissmodel.expasy.org.

The UCSF Chimera software was used for molecular graphics, and surfaces were created with the MSMS package [63].

Docking analyses were performed with the *L. albus* γC model and β-mannosidase with the best resolution in the PDB repository, namely, the enzyme from *T. harzianum* ThMan2A (PDB: 4CVU) [55]. These elaborations were performed with different on-line available software: pyDockWEB [64], ClusPro 2.0 [65], PRISM 2.0 [66,67], GRAMM-X Protein-Protein Docking Web Server v.1.2.0 [68], HDOCK Server [69] and FRODOCK [70]. All the tools used compute protein–protein interaction using Fast Fourier Transform and rigid-body structural matching, followed by the refinement of the predicted complexes and global energy calculation. The docking of the substrate galactomannan (PubChem CID: 439336) into the enzyme’s active site was performed with AutoDock Vina, available from UCSF Chimera [71].

The Metal Ion-Binding site prediction and docking server (MIB) was used to analyze the potential Zn^2+^ binding sites of γC. This tool [72] takes advantage of the fragment transformation method for structural comparison between query proteins and templates, after the selection and the gathering of ion binding residues into the query 3D structure and those within 3.5 Å of the metal ion [73,74].

Glycosylation was added to the γC model using the Glyprot software [75], available as web-server tool [76], which correctly identified the glycosylation residue N131 in the γC sequence.

### 3.7. Statistical Analysis

All determinations were carried out in triplicate. Data reported in the histograms are expressed as the means ± S.E. Data were analyzed by *t*-tests. *p* values < 0.05 were considered to be statistically significant.

## 4. Conclusions

In this work, we first describe an inhibitory activity of *L. albus* γC, an effect sought for a long time in vain. Considering its homology with XEGIPs, attention has been previously focused on investigating its activity towards those enzymes targeted by XEGIPs themselves. However, switching the target towards enzymes potentially more relevant to legumes’ cell wall attacks, namely, enzymes acting on β-galactomannan degradation, enabled us to obtain first insights into possible γC involvement in cellular responses to pathogens, strengthening the possibility that γC is a multi-functional protein. From an evolutionary point of view, the production and accumulation in seeds of huge amounts of a particular protein that would be needed only if certain microbes attacked, is unfavorable. Normally, antimicrobial compounds are conditionally expressed [77]. Thus, a combination of storage and antimicrobial roles for γC is plausible, as described for some seed storage proteins [78] and considering that γC is one of the last seed storage proteins to be degraded during germination [79].

In silico predictions showed that the interaction of γC with GH2 β-mannosidase can occur in proximity to the active site and allowed us to propose that the potential inhibitory activity could be mediated by structures that are peculiar to γC, given the lack of XEGIPs’ IL1 and IL2 characteristics. These findings are consistent with the experimental activities since a stoichiometry of 1:1 can be hypothesized from the titration curve of GH2 β-mannosidase with γC. Interestingly, this inhibitory activity described is mediated by the presence of a metal ion. This finding raises new questions on the inhibitory mechanisms of GHIPs, in particular, those that, to date, have been identified as homologs but have failed to demonstrate inhibition, for example, soybean Bg7S. The role of Zn^2+^ in enhancing the inhibitory activity of γC deserves further research.

By and large, this work describes experimental findings that highlight interesting new scenarios for understanding the natural role of γC. Although structural predictions can leave space for speculative interpretations, the full set of data reported in this work allows one to hypothesize possible and allows one to hypothesize possible mechanisms of action for the basis of inhibition. As a matter of fact, at least two mechanisms seem plausible, both involving elements that are peculiar to the lupin γC structure.

## Supplementary Materials

Supplementary materials can be found at https://www.mdpi.com/1422-0067/21/19/7305/s1. Supplementary Figure S1: Surface localization of one of the putative Zn2+ binding site to conglutin gamma, Figure S2: Structure superposition of ThMan2A (cyan) with GH2 model (tan) created by Swiss model based on the amminoacid sequence of *Cellulomonas fimi* GH2 used in in vitro experiments (Panel A). Close up of the active site with catalytic glutammic acids residues highlighted in stick view (Panel B). RMSD of the two matched structures is 0.987 Å.

## Abbreviations

GH	Glycoside hydrolase
GHIPs	Glycoside hydrolase inhibitor proteins
GM	β-galactomannan
GMQE	Global model quality estimation
IL1	Inhibitory loop 1
IL2	Inhibitory loop 2
pNP-β-d-mannopyranoside	para-nitrophenyl-β-d-mannopyranoside
QMEAN	Qualitative model energy analysis
RMSD	Root mean square deviation
