# Supplementary material for: Lupinus albus γ-Conglutin, a Protein Structurally Related to GH12 Xyloglucan-Specific Endo-Glucanase Inhibitor Proteins (XEGIPs), Shows Inhibitory Activity against GH2 β-Mannosidase
Source: Int J Mol Sci. 2020 Oct 3;21(19):7305. doi: 10.3390/ijms21197305 (PMC7583008; doi:10.3390/ijms21197305)
Supplement: Supplementary file 1 [file ijms-21-07305-s001.zip › Supplementary Figure S1.pdf]

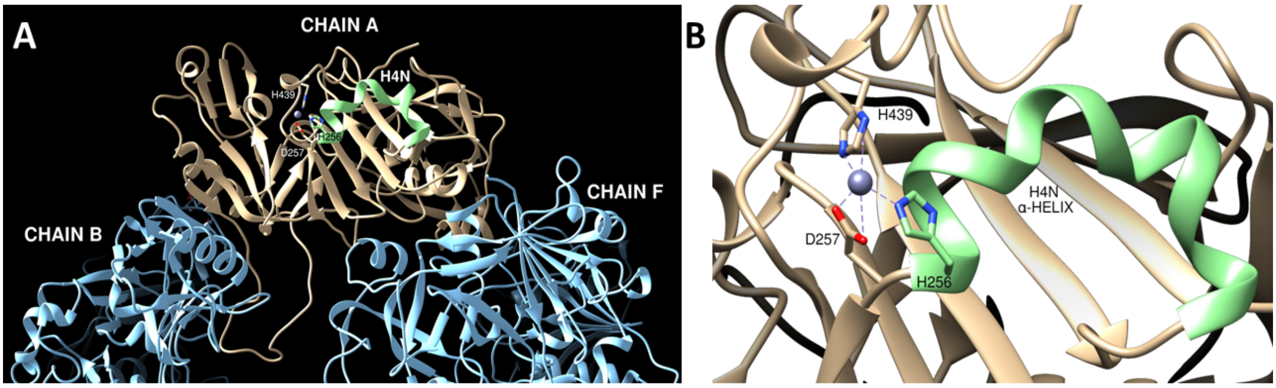

Supplementary Figure S1. Surface localization of one of the putative Zn2+ binding site to conglutin gamma. Panel A shows that the interested region is solvent exposed even in the whole assembled hexameric structure. Panel B shows the magnification of the putative zinc coordination site. Zn2+ atom is shown in grey; coordinating residues H439, H256 and D257 are displayed in stick representation; H4N α-helix [21] is colored in light green. Monomer A of γC is tan colored, while the other monomers forming the quaternary structure are cyan colored. The identification of amino acid residues of γC potentially involved in zinc coordination was carried out resorting to Metal Ion-Binding Site Prediction and Docking Server (MIB). The predictions identified a region including the inhibitory loop IL2, also present in GH11 and GH12 inhibitors, namely TAXI-I and XEGIP [39]. Within IL2, residue H439 is of key importance as demonstrated earlier in a recombinant mutant version of γC [20]. This histidine is conserved in TAXI-I IL2, but in XEGIP the same position is occupied by an arginine residue.
