# Supplementary material for: Lupinus albus γ-Conglutin, a Protein Structurally Related to GH12 Xyloglucan-Specific Endo-Glucanase Inhibitor Proteins (XEGIPs), Shows Inhibitory Activity against GH2 β-Mannosidase
Source: Int J Mol Sci. 2020 Oct 3;21(19):7305. doi: 10.3390/ijms21197305 (PMC7583008; doi:10.3390/ijms21197305)
Supplement: Supplementary file 1 [file ijms-21-07305-s001.zip › Supplementary Figure S2.pdf]

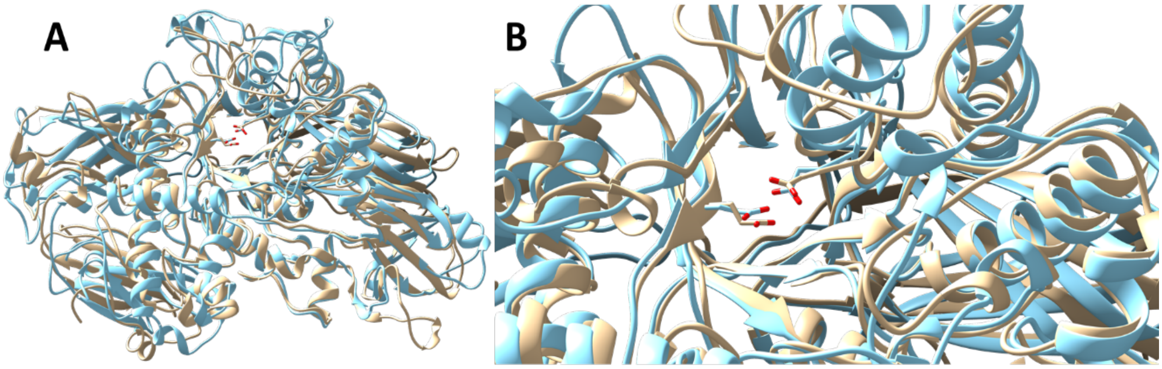

Supplementary Figure S2. Structure superposition of ThMan2A (cyan) with GH2 model (tan) created by Swiss model based on the amminoacid sequence of *Cellulomonas fimi* GH2 used in in *vitro* experiments (Panel A). Close up of the active site with catalytic glutammic acids residues highlighted in stick view (Panel B). RMSD of the two matched strucures is 0.987 Å.
